# Gender disparities in depression severity and coping among people living with HIV/AIDS in Kolkata, India

**DOI:** 10.1371/journal.pone.0207055

**Published:** 2018-11-21

**Authors:** Dallas Swendeman, Anne E. Fehrenbacher, Soma Roy, Rishi Das, Protim Ray, Stephanie Sumstine, Toorjo Ghose, Smarajit Jana

**Affiliations:** 1 Department of Psychiatry & Biobehavioral Sciences, David Geffen School of Medicine, University of California, Los Angeles, California, United States of America; 2 Center for HIV Identification, Prevention & Treatment Services (CHIPTS), University of California, Los Angeles, California, United States of America; 3 Center of Expertise in Women’s Health, Gender, and Empowerment, University of California Global Health Institute, San Francisco, California, United States of America; 4 Sonagachi Research & Training Institute, Durbar Mahila Samanwaya Committee, Kolkata, India; 5 School of Social Policy and Practice, University of Pennsylvania, Philadelphia, Pennsylvania, United States of America; CSOM, UNITED STATES

## Abstract

People living with HIV/AIDS (PLH) experience high rates of depression and related psychosocial risk factors that vary by gender. This study examines gender differences in depression severity among antiretroviral therapy (ART) patients (n = 362) from a large government ART clinic in Kolkata, India. Hypotheses for multiple linear regression models were guided by an integrated gendered stress process model focusing on variables reflecting social status (age, partner status), stressors (stigma), and resources (income, social support). Depressive symptoms were assessed with the Hospital Anxiety and Depression Scale (HADS); 22% of the sample reached the cutoff for severe depression, 56% moderate, and 13% mild depression. Compared to men, women reported lower income, education (50% no formal education vs. 20% men), availability of emotional and instrumental support, and were less likely to be married or cohabiting (53% women vs. 72% of men). However, more women had partners who were HIV-positive (78% women vs. 46% men). Overall, depression severity was negatively associated with availability of emotional support and self-distraction coping, and positively associated with internalized HIV/AIDS stigma, availability of instrumental support, and behavioral disengagement coping. Interactions for instrumental support by income and partner status by age varied significantly by gender. Analyses stratified by gender indicated that: 1) Frequently seeking instrumental support from others was protective for men at all income levels, but only for high-income women; and 2) having a partner was protective for men as they aged, but not for women. These results suggest that gender disparities in depression severity are created and maintained by women’s lower social status and limited access to resources. The effect of stigma on depression severity did not vary by gender. These findings may inform the tailoring of future interventions to address mental health needs of PLH in India, particularly gender disparities in access to material and social resources for coping with HIV.

**Trial Registration:** ClinicalTrials.gov registration #NCT02118454, registered April 2014.

## Introduction

India has the third largest population of people living with HIV/AIDS (PLH) in the world, estimated at 2.3 million [1]. Depression is one of the most common co-morbidities of HIV worldwide [2]. High prevalence of depression has consistently been documented among PLH in India [3–8]. The few studies that have examined gender differences in depression among PLH in India have found higher prevalence and severity of depression among women than men [9, 10]. However, previous studies on HIV and mental health in India have included small samples of PLH (n < 100), which limit meaningful comparisons between men and women.

No studies to our knowledge have examined whether the relationship between gender and depression among PLH in India is modified by disparities between men and women in social status, exposure to stressors, or access to resources. For example, internalized HIV/AIDS stigma is a common stressor for PLH, and the effect of stigma on depression might vary by gender [11]. Similarly, access to and use of social support resources can modify the relationship between gender and depression [12].

This paper presents results from a cross-sectional analysis of baseline data with a sample of PLH (n = 362) at a large government clinic in Kolkata, India engaged in a randomized controlled trial of mobile phone support for ART adherence and self-management over six months [3]. First, we examined rates of mild, moderate, and severe depressive symptomology. Second, we tested gender differences in associations between depression severity and social status, stigma, and coping resources. We proposed a novel theoretical model, which posits that gender disparities in depression severity among PLH in India are maintained and exacerbated by an unequal distribution of stressors, resources, and social status between men and women.

### Theoretical framework: Integrated model of the gendered stress process

**Fig 1:**
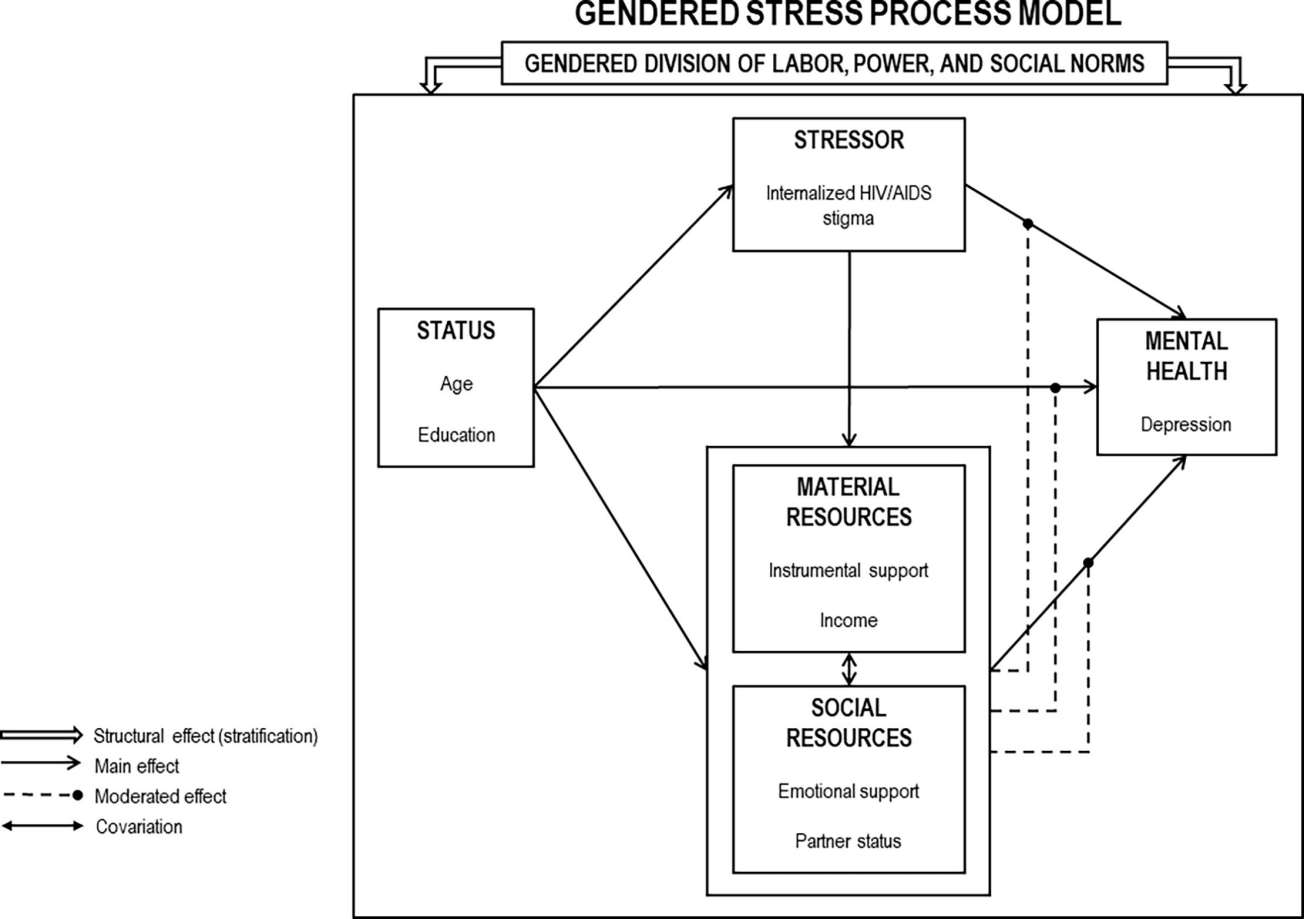
Gendered stress process model.

To examine gender differences in depression among PLH, we designed a model integrating the theory of gender and power [13] with the stress process model [14] (Fig 1). The theory of gender and power explains how social and institutional structures contribute to gender imbalances in power [13]. The stress process model explains how mental health disparities are created and maintained by social stratification, such that people at the bottom of the social hierarchy are exposed to more stressors and have access to fewer resources for coping with those stressors [14].

According to the theory of gender and power, three distinct but overlapping structures simultaneously create gender inequities between men and women: the gendered division of labor, the gendered division of power, and the structure of cathexis, which refers to social norms [15]. The unequal divisions of labor, power, and social norms create gender disparities, such as occupational segregation, less control over resources, and lower expectations for women compared to men in institutions, such as schools and workplaces [13]. In turn, these disparities lead to environmental exposures, interpersonal risks, and biological vulnerabilities that increase women’s susceptibility to disease and violence [16, 17]. The theory of gender and power has been applied to investigate disparities in HIV risk and physical outcomes, but it remains underutilized in the study of HIV and mental health [15, 18].

The stress process model explains how a person’s status within a stratified society influences exposure to stressors and access to resources, which in turn, create and sustain mental health disparities [14]. We theorize that the unequal status of men and women in India and the large gender gap in access to resources make the stress process of receiving an HIV diagnosis and managing ART adherence more detrimental to mental health for women than men.

### Study hypotheses

Based on this integrated model of the gendered stress process, we hypothesize that the relationship between gender and depression severity among PLH in India is conditional on social statuses, stressors, and coping resources (See Fig 1). In the model, we conceptualize gender, age, and partner status as *social status* characteristics, stigma as a *stressor*, and emotional support, instrumental support, and income as *resources*. Partner status functions as both a *social status* and a *social resource*. Emotional support is a *social resource* while instrumental support and income are *material resources*. Hypotheses for the conditional relationships in the model are outlined below:

**Conditional effect of social statuses:** Age X partner status
**H1-A:** Age will be positively associated with depression level for both men and women due to accelerated functional decline from HIV with aging.
**H1-B:** Having a partner will reduce the strength of the relationship between age and depression level more for men than women. We expect that men will benefit more from having a partner as they age because women in India are expected to take on the role of caregiver for their spouses, but men are not [19].
**Conditional effect of stressors:** Stigma X partner status
**H2-A:** Internalized HIV/AIDS stigma will be positively associated with depression level for both men and women. In India, women with HIV/AIDS experience stigma due to assumptions about infidelity or engagement in sex work, whereas men experience stigma primarily due to assumptions about being gay, or to a lesser extent, purchasing commercial sex [20–22]. We expect the relationship between stigma and depression level to be stronger for women than men because the stigma experienced by PLH in India is more likely to be accompanied by physical abuse, social ostracism, and loss of economic resources for women than men [23–25].
**H2-B:** Having a partner will reduce the strength of the relationship between stigma and depression more for men than women. We expect that the positive relationship between stigma and depression level will be dampened more for men with a partner than women with a partner because women in India are expected to provide more emotional and caregiver support for sick spouses than men are [26–28]. Additionally, men living with HIV in India are more likely to be married to their partners than women living with HIV in India and are less likely to experience marital dissolution or have a partner desert them as a result of their diagnosis [29].
**Conditional effect of resources:** Instrumental support X income
**H3-A:** Instrumental support will be negatively associated with depression level for both men and women. We expect the protective effect of instrumental support to be stronger for men than women. Although men have access to more material resources than women in India, many studies suggest that women still provide more instrumental support to others than men provide on average, when someone close to them seeks financial or other forms of tangible assistance [30, 31].
**H3-B:** We expect that the relationship between instrumental support and depression severity will be conditional on income, such that higher income people will experience a stronger protective effect of instrumental support on depression level because they are more likely to be in social networks with other high income people who are capable of providing material support without eroding scarce resources or damaging social ties. In India, men and women have large income disparities, in which women are severely disadvantaged economically, so we expect the protective effect of coping by drawing on instrumental support by income level to be stronger for men than women.

Together, lower social status, higher exposure to stigma, and lower access to social support and economic resources among women compared to men in India will explain gender disparities in depression severity among PLH in India.

## Methods

### Study recruitment and data collection

Participants were recruited primarily from the Calcutta School of Tropical Medicine (STM) ART clinic, one of the three primary government sponsored ART clinics in Kolkata, India. Secondary recruitment was conducted from the Mamata Care and Treatment Center (MCTC), which is an ancillary support services center serving HIV-positive sex workers and their families. Recruitment was facilitated through verbal introduction scripts to patients in waiting areas of STM and MCTC, followed by the administration of screening questions in private.

Eligibility criteria were: 1) age of 18 years or older; 2) duration on ART of at least six months; 3) on first or second line ART (i.e., not on third line therapy due to prior treatment failures); 4) availability of CD4 counts in the prior two months (conducted routinely at six-month intervals); and 5) self-reported missing at least one ART dose in the past six months. If a prospective participant was eligible based on self-report, eligibility was confirmed by study staff reviewing the participant's “ART Cards” (i.e., a medical record issued by physicians to all ART patients in India). Signed consent forms were stored in a locked file in a locked office. Of 384 patients screened from April to July 2014, 362 were eligible and consented to participate in the study. Interviews were conducted in Bengali (primarily), Hindi, or English based on participants’ language preferences.

Participants were interviewed by a trained research team member administering a face-to-face baseline interview using a mobile phone application to record responses. Participants were queried on depression symptoms, social support, HIV-status disclosures, sexual behaviors, alcohol use, HIV/AIDS stigma, and strategies for coping with HIV/AIDS and ART adherence. Written informed consent was obtained from all individual participants included in the study. The protocol for this study was approved by the Institutional Review Boards of the University of California, Los Angeles and the Durbar Ethical Review Board. All procedures performed involving human participants were in accordance with the ethical standards of the Institutional Review Boards of University of California, Los Angeles and the Durbar Ethical Review Board in West Bengal, India.

### Measures

#### Depression

The Hospital Anxiety and Depression Scale (HADS) was utilized to measure depression through the 8-item depression sub-scale (HADS-D). The HADS instrument has been validated in psychiatric research globally, with good internal validity and external consistency relative to more burdensome diagnostic tools such as ICD-08 and DSM-IV [32–34]. Example questions include, “I still enjoy the things I used to enjoy,” “I feel as if I am slowed down,” and “I have lost interest in my appearance”. Responses on a Likert-scale range from 0 “Not at all” to 3 “Most of the time” and reverse coded for negatively-framed items. The HADS-D has four recommended scoring levels based on the sum of the responses: none (0–7), mild (8–10), moderate (11–14), severe (15+). In this study, the HADS-D score was treated as a continuous variable indicating severity of depressive symptoms for linear regression analyses.

A recent pilot study in Kolkata, India validated the brief HADS measures of depression and anxiety with PLH [35] that was translated in Bengali to reduce confounding from somatization of physical symptoms and functional impairment. A second pilot study with PLH for the current study using this measure found high rates of depression and low rates of anxiety [3]. Therefore, the anxiety subscale was excluded in this study to reduce assessment burden.

#### Modified medical outcomes study social support (mMOS-SS) brief measures

The mMOS-SS operationalizes the perceived availability of emotional (intangible) support and instrumental (tangible) support. This brief 8-item version demonstrates excellent reliability, sensitivity, and specificity for two higher order factors of emotional support (caring, loving, and empathy) and instrumental support (tangible assistance and material resources provided by others) [36].

#### Coping with HIV and taking ART: Brief COPE measures

The Brief COPE is a reliable and abbreviated version of the longer COPE inventory, which has been used to assess frequency of behaviors for coping with chronic illnesses [37–39]. For this study, the questions were framed to assess coping specifically with HIV and taking ART. There are 14 sub-scales consisting of two items each with responses ranging from 0 “Not doing this at all” to 3 “Doing this a lot” and summed to a 0–6 score for each sub-scale domain: self-distraction, active coping, denial, substance use, use of emotional support, use of instrumental support, behavioral disengagement, venting, positive reframing, planning, humor, acceptance, religion, and self-blame. For example, self-distraction is assessed with the items: “I've been turning to work or other activities to take my mind off things” and “I've been doing something to think about it less, such as going to movies, watching TV, reading, daydreaming, sleeping, or shopping”.

#### Stigma

Internalized HIV/AIDS Stigma is a six-item measure of self-effacing and negative self-perceptions endorsed by PLH. The measure has been validated with ART patients in global contexts [40]. Items included: “It is difficult to tell people about my HIV infection,” and “Being HIV positive makes me feel dirty,” with “agree” or “disagree” responses to each item and summed to a 0–6 scale.

#### Alcohol use

AUDIT-C is an abbreviated 3-item alcohol screening scale, derived from the 10-item AUDIT instrument, and designed to identify hazardous drinkers or individuals with active alcoholism disorders. AUDIT-C is scored on a scale of 0–12 by summing responses to the three items. A higher score is associated with hazardous drinking habits and has been shown to be a reliable, practical, and valid measure to assess heavy drinking and alcohol use [41, 42].

#### Demographic characteristics

Characteristics of study participants included as control variables or moderators in the analysis were gender, age, income, educational attainment, partner status, HIV status of partner, and number of dependents. Gender is a dichotomous variable with responses 1 “Woman” versus 0 “Man”. Two transgender women were included in the “Woman” category since they identified as women and they mirrored cisgender women in the study on all measured demographic variables and not the cisgender men. Age is a continuous variable measured in years. Income is a continuous variable of individual monthly income categorized in 100 Indian rupee (INR) increments for analyses. Educational attainment is an ordinal categorical variable measured as highest education level completed with responses 0 “No formal education and illiterate”, 1 “No formal education but literate”, 2 “Class 5”, 3 “Class 10”, 4 “Class 12”, 5 “Graduate”, and 6 “Post-graduate”. Partner status is a dichotomous variable with responses 0 “Single and living alone” versus 1 “Married or living with partner”. HIV status of partner is a dichotomous variable with responses 0 “HIV-negative” versus 1 “HIV-positive or not sure”. Number of dependents is a continuous variable for total number of people relying on the respondent’s financial support ranging from zero to ten people.

### Statistical data analysis

Baseline descriptive analysis was conducted using simple univariate frequency distribution methods. Bivariate correlation tables were used to inform variable selection and model building for the multiple linear regression analysis. Depression severity was analyzed as a continuous outcome variable ranging from 0–24 using multiple linear regression. Gender was used as the primary independent variable and is the main component of interactions in the multiple linear regression model. All analyses were conducted in Stata 13.1.

A hypothesis-driven approach was utilized to select predictors that had a theoretical basis for inclusion into multiple linear regression models either as a hypothesized predictor from the integrated gendered stress process model or as a control variable for confounding. Next, second-order interactions of gender by age, partner status, stigma, emotional support, and instrumental support were added to the models. Then, third-order interactions for gender by age by partner status and gender by instrumental support by household income were added. Because both third-order interactions were significant, we then stratified the multiple linear regression models by gender and re-tested correlates of depression severity separately for men and women to facilitate interpretation of the interactions.

Following an initial screen for collinearity of variables, two automated regression procedures were utilized as a sensitivity analysis to identify a best fit model using subset regression and score procedures. The subset and score methods resulted in highly concordant models with the same covariates being selected by the hypotheses for both models of the same size. Model fit was assessed in the linear regression models by comparisons of Akaike information criterion (AIC) values and the adjusted R^2^ value.

There were a small number of missing values for partner status (1 missing value) and income (24 missing values). All other variables had complete data. A sensitivity analysis using multiple imputation was conducted and yielded the same findings as the complete case analysis. For ease of interpretation, we present only the results from the complete case analysis in the tables and graphs of interactions.

## Results

Table 1 provides a summary of sample characteristics overall and stratified by gender. The majority of participants were men with an average age of 39 years. Men in the sample had significantly higher social status across several demographic variables compared with women, consistent with gender disparities in the Indian general population. Men had higher income and educational attainment, were older, had more dependents, were more likely to be married or living with a partner, and were less likely to have a partner who was also living with HIV than women (all p<0.001). Approximately three-quarters of men in the sample were married or cohabiting compared with only half of the women. Nearly 80% of women in the sample had a partner who was also HIV-positive compared with less than half of men. Half of the women had no formal education compared with 20% of the men. Mean income was 5,184 Rs/month, with men reporting more than double the income of women, on average (6,459 Rs vs. 2,541 Rs, p < .001).

**Table 1:** Sample characteristics of people living with HIV in India, stratified by gender (N = 362).

	Women (n = 124)		Men (n = 238)		Total (N = 362)		P-value
	Freq. \ Mean	% \ SD	Freq. \ Mean	% \ SD	Freq. \ Mean	% \ SD	
**Dependent Variable**	** **	** **	** **	** **	** **	** **	** **
Depression Severity (HADS-D) [0–24] (Mean, SD)	13.0	2.9	12.3	3.7	12.5	3.5	0.076†
None (0–7)	5	4.1	28	11.8	33	9.1	
Mild (8–10)	15	12.3	30	12.6	46	12.7	
Moderate (11–14)	73	59.8	130	54.6	204	56.4	
Severe (15–24)	29	23.8	50	21.0	79	21.8	
**Independent Variable**	** **	** **	** **	** **	** **	** **	** **
Gender (%)	124	34.3	238	65.7	362	100	n.a.
**Moderator Variables**	** **	** **	** **	** **	** **	** **	** **
*Status*							
Age in Years [22–65] (Mean, SD)	36.7	8.0	40.6	8.7	39.2	8.6	< .001***
*Stressor*							
Internalized HIV/AIDS Stigma [0–6] (Mean, SD)	2.6	1.5	2.6	1.8	2.6	1.7	0.975
*Resources*							
Married or Living with Partner (%)	64	52.5	171	71.5	234	64.8	< .001***
Monthly Income in Indian Rupees [0–60,000] (Mean, SD)	2540.9	3054.0	6458.8	7395.0	5184.0	6575.0	< .001***
Frequency of Instrumental Support (Brief-COPE) [0–6) (Mean, SD)	2.2	1.3	2.4	1.3	2.4	1.3	0.135
**Control Variables**							
Educational Attainment by Highest Grade [0–6] (Mean, SD)	1.5	1.3	2.4	1.4	2.1	1.4	< .001***
No Formal Education, Illiterate (0)	36	29.5	26	10.9	62	17.1	
No Formal Education, Literate (1)	29	23.8	20	8.4	49	13.5	
Class 5 (2)	28	23.0	91	38.2	119	32.9	
Class 10 (3)	22	18.0	64	26.9	86	23.8	
Class 12 (4)	6	4.9	18	7.6	25	6.9	
Graduate (5)	1	0.8	14	5.9	16	4.4	
Post Graduate (6)	0	0.0	5	2.1	5	1.4	
Has an HIV+ Partner (%)	50	78.1	79	46.2	129	55.1	< .001***
Number of Dependents (Mean, SD)	1.4	1.6	2.7	1.8	2.2	1.9	< .001***
Alcoholism (AUDIT-C) [0–12] (Mean, SD)	0.3	1.0	0.5	1.3	0.4	1.2	0.072†
Availability of Social Support (mMOS-SS) [0–100] (Mean, SD)							
Instrumental Support	38.4	34.2	58.5	36.1	51.6	37.0	< .001***
Emotional Support	30.3	26.0	42.5	26.7	38.3	27.0	< .001***
Frequency of Coping Responses (Brief-COPE) [0–6] (Mean, SD)							
Behavioral Disengagement	1.0	1.3	1.8	1.5	1.5	1.5	0.000***
Active Coping	2.6	1.1	3.1	1.3	2.9	1.2	0.000***
Self-Blame	0.8	1.0	1.2	1.3	1.1	1.2	0.003**
Planning	2.1	1.1	2.5	1.1	2.4	1.1	0.004**
Venting	1.3	1.3	1.8	1.5	1.6	1.5	0.005**
Acceptance	3.3	1.7	3.8	1.7	3.7	1.7	0.006**
Humor	0.6	0.9	0.4	0.8	0.4	0.8	0.005**
Substance Abuse	0.1	0.3	0.3	0.8	0.2	0.7	0.021**
Denial	0.8	0.9	1.1	1.3	1.0	1.2	0.016*
Positive Reframing	2.5	1.2	2.7	1.3	2.7	1.3	0.043†
Self-Distraction	2.5	1.3	2.8	1.5	2.7	1.4	0.059†
Emotional Support	2.3	1.3	2.4	1.4	2.4	1.3	0.511
Religion	1.6	1.5	1.4	1.6	1.5	1.5	0.266

Notes: Significance level

† = p<0.10

* = p<0.05

** = p<0.01

*** = p<0.001

All scales ordered low to high

Monthly income is per 100 Indian Rupees

HADS-D is a subscale of the Hospital Anxiety and Depression Scale used to determine level of depression severity Brief-COPE is an abbreviated version of the COPE Inventory used to assess frequency of coping responses

AUDIT-C is an alcohol screening tool to identify hazardous drinkers or active alcohol use disorders.

mMOS-SS is the modified Medical Outcomes Study Social Support Survey used to measure availability of emotional and instrumental support.

Prevalence of depressive symptoms was high overall, with 56% of respondents scoring in the moderate range and 22% in the severe range. There was a marginal trend for gender differences in depression level (p = 0.08) but not a significant difference in the unadjusted bivariate comparison. AUDIT-C scores were low overall with mean score of 0.4 on a scale of 0–12. Internalized HIV/AIDS stigma scores were moderate overall with mean score of 2.6 on a 0–6 scale.

Overall, perceived availability of instrumental support was higher compared to perceived availability of emotional support (mean mMOS-SS score of 52 vs. 38 on 0–100 scale), and women reported lower availability of social support than men for both subscales (p < .001). The most common coping strategies used for dealing with HIV/AIDS and ART adherence were acceptance, active coping, self-distraction, positive reframing, planning, and drawing on emotional and instrumental support. Compared to women, men reported higher frequency of using active coping, acceptance, self-blame, substance abuse, denial, behavioral disengagement, venting, and planning. Men used humor to cope less frequently than women.

### Multiple linear regression for predictors of depression severity

Table 2 presents the unstratified multiple linear regression model for predictors of depression severity as a continuous outcome. The model explained 52% of the variance in depression severity. Overall, depression severity was negatively associated with availability of emotional support from others (B = -0.06, 95% CI: -0.08 –-0.04) and frequency of using self-distraction as a coping strategy (B = -1.08, 95% CI: -1.34 –-0.82). Depression level was positively associated with internalized HIV/AIDS stigma (B = 0.61, 95% CI: 0.12–1.11), availability of instrumental support from others (B = 0.03, 95% CI: 0.01–0.04), and frequency of using behavioral disengagement as a coping strategy (B = 0.43, 95% CI: 0.22–0.65).

**Table 2:** Unstandardized multiple linear regression coefficients and 95% confidence intervals for correlates of depression severity among people living with HIV in India (n = 337).

Depression Severity	*B*	95% CI	
Constant	13.992	10.584	17.401
Gender	-0.462	-4.843	3.919
Age	-0.026	-0.100	0.047
Married or Living with Partner	-4.348†	-9.178	0.482
Income (per 100 INR)	0.022	-0.006	0.050
Internalized HIV/AIDS Stigma (0–6)	0.612*	0.120	1.105
Availability of Emotional Support (0–100)	-0.062***	-0.084	-0.039
Availability of Instrumental Support (0–100)	0.029***	0.012	0.044
Frequency of Instrumental Support (0–6)	0.731**	0.207	1.254
Frequency of Disengagement (0–6)	0.433***	0.220	0.646
Frequency of Self-Distraction (0–6)	-1.081***	-1.343	-0.819
Gender X Frequency of Instrumental Support	-1.343***	-1.933	-0.753
Gender X Income	-0.035*	-0.065	-0.005
Frequency of Instrumental Support X Income	-0.015*	-0.029	-0.002
Gender X Frequency of Instrumental Support X Income	0.019**	0.005	0.032
Gender X Age	0.078	-0.021	0.176
Gender X Married or Living with Partner	8.571**	2.686	14.457
Married or Living with Partner X Age	0.134*	0.008	0.260
Gender X Married or Living with Partner X Age	-0.217**	-0.365	-0.068
F-test	F(21, 315)	17.91***	
Adjusted R-squared	0.51		

Notes: Significance level

† = p<0.10

* = p<0.05

** = p<0.01

*** = p<0.001

B = Regression coefficient

CI = Confidence interval

Among the coping strategies, self-distraction was associated with lower depression levels and behavioral disengagement was associated with higher depression levels. Specifically, for every one-unit increase on the COPE self-distraction scale (range 0–6), depression level decreased by 1.08 points, whereas for every one-unit increase on the COPE behavioral disengagement scale (range 0–6), depression level increased by 0.43 points. For every one-unit increase in internalized HIV/AIDS stigma, depression level increased by 0.61 points. For every 10-point increase in perceived availability of emotional support (range 0–100), depression level decreased by 0.06 points, and for every 10-point increase in perceived availability of instrumental support, depression level increased by 0.03 points.

Two of the three hypothesized interactions based on the gendered stress process model were significantly associated with depression level. The significant interactions were gender X frequency of coping by drawing on instrumental support X income (B = 0.02, 95% CI: 0.01–0.03) and gender X partner status X age (B = -0.22, 95% CI: -0.37 –-0.07). The interaction of gender X partner status X internalized HIV/AIDS stigma was tested but was not significantly associated with depression level (B = 0.01, 95% CI: -0.74–0.77, p = 0.973), so it was not included in Tables 2 and 3. These results support the hypotheses that gender disparities in depression are created and maintained by women’s lower social status and access to resources, but not the hypothesis regarding gender differences in exposure to stigma as a stressor.

**Table 3:** Unstandardized multiple linear regression coefficients and 95% confidence intervals for correlates of depression severity among people living with HIV in India, stratified by gender (n = 337).

	Women (n = 109)			Men (n = 228)		
Depression level	*B*	95% CI		*B*	95% CI	
Constant	13.989***	10.825	17.154	13.584***	10.793	16.375
Age	-0.023	-0.092	0.046	0.052	-0.016	0.120
Married or living with partner	-4.374*	-8.641	-0.107	4.178*	0.934	7.421
Income (per 100 INR)	0.021	-0.005	0.047	-0.013*	-0.023	-0.003
Internalized HIV/AIDS stigma (0–6)	0.587***	0.288	0.886	0.399***	0.207	0.591
Availability of emotional support (0–100)	-0.081***	-0.118	-0.045	-0.051***	-0.080	-0.023
Availability of instrumental support (0–100)	0.049***	0.022	0.077	0.019†	-0.001	0.039
Frequency of instrumental support (0–6)	0.720*	0.170	1.270	-0.645**	-1.050	-0.240
Frequency of disengagement (0–6)	0.405†	-0.051	0.860	0.443***	0.199	0.688
Frequency of self-distraction (0–6)	-1.187***	-1.700	-0.675	-1.051***	-1.358	-0.744
Frequency of instrumental support X Income	-0.015*	-0.027	-0.002	0.003†	-0.001	0.007
Married or living with partner X Age	0.133*	0.015	0.251	-0.084*	-0.166	-0.003
F-test	F (12, 96)	7.78***		F(12, 215)	22.420***	
Adjusted R-squared	0.429			0.532		

Notes: Significance level

† = p<0.10

* = p<0.05

** = p<0.01

*** = p<0.001

B = Regression coefficient

CI = Confidence interval

### Multiple linear regression for predictors of depression severity stratified by gender

Table 3 presents the multiple linear regression coefficients and 95% confidence intervals for covariates of depression severity, stratified by gender. The model for women explained 43% of the variance in depression severity among women. The model for men explained 53% of the variance in depression severity among men. Among women, depression severity was negatively associated with availability of emotional support (B = -0.08, 95% CI: -0.12 –-0.05) and frequency of coping by self-distraction (B = -1.19, 95% CI: -1.70 –-0.68) and positively associated with internalized HIV/AIDS stigma (B = 0.59, 95% CI: 0.29–0.89) and availability of instrumental support (B = 0.05, 95% CI: 0.02–0.08). Like women, depression severity among men was negatively associated with availability of emotional support (B = -0.05, 95% CI: -0.08 –-0.02) and frequency of coping by self-distraction (B = -1.05, 95% CI: -1.36 –-0.74) and positively associated with internalized HIV/AIDS stigma (B = 0.40, 95% CI: 0.21–0.59). In contrast to women, depression severity among men was negatively associated with frequency of drawing on instrumental support (B = -0.65, 95% CI: -1.05 –-0.24) and positively associated with frequency of coping by behavioral disengagement (B = 0.44, 95% CI: 0.20–0.69).

**Fig 2:**
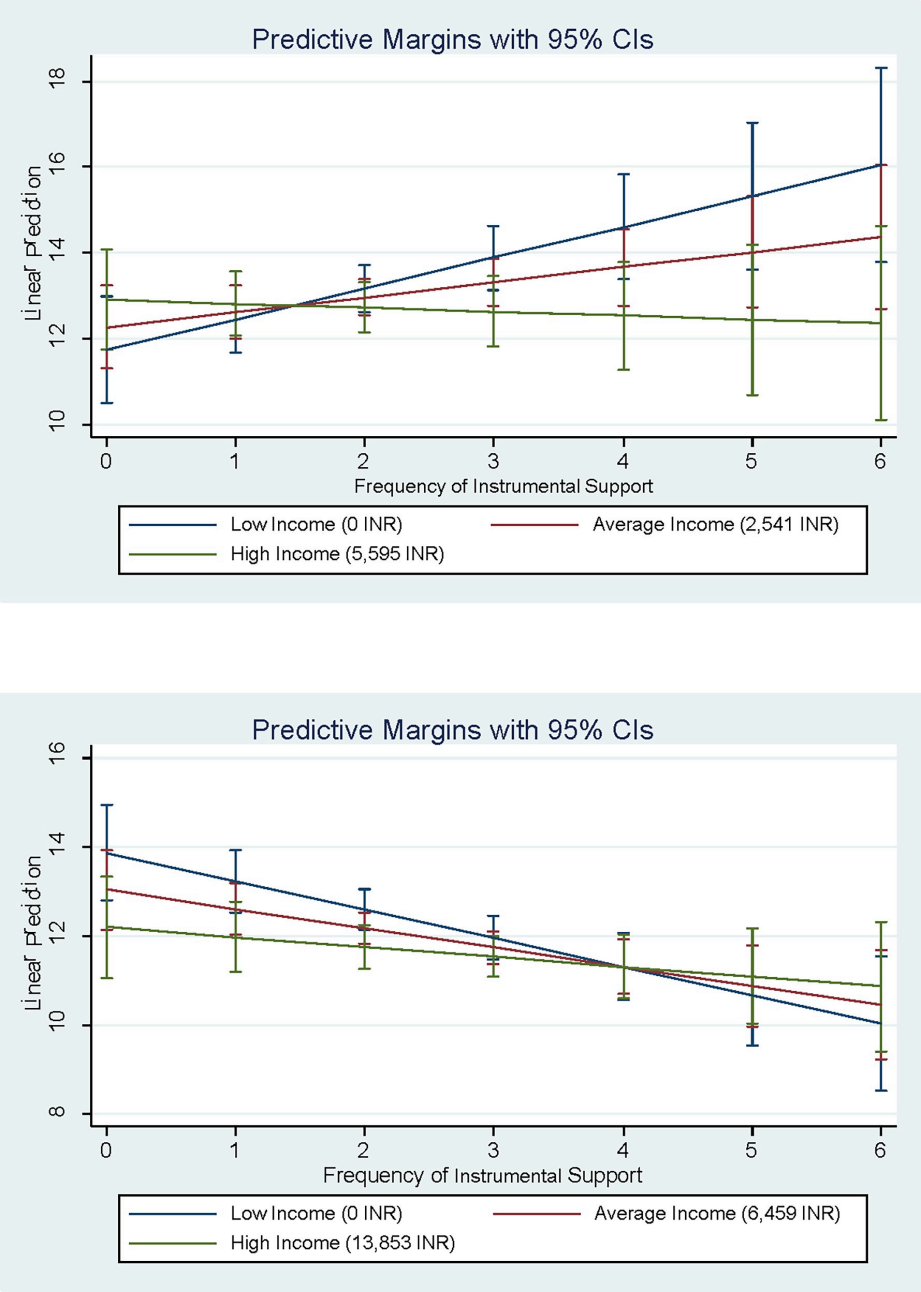
a)Interaction of Instrumental Support by Income for Women b) Interaction of Instrumental Support by Income for Men.

The interaction of frequency of coping by drawing on instrumental support X income was significantly associated with depression severity for women but not for men (see Fig 2A and 2B). For men of all income levels, frequency of drawing on instrumental support to cope with HIV was associated with lower severity of depression. However, this association was observed only for high-income women. Instrumental support was associated with higher severity of depression for women low-income women.

**Fig 3:**
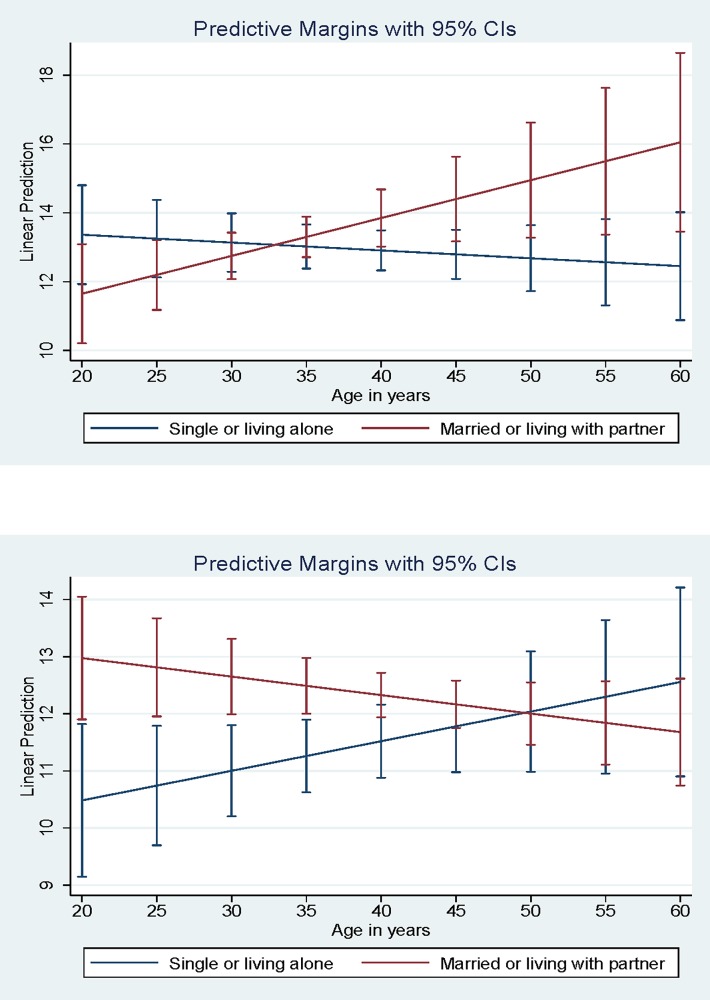
**a) Interaction of Partner Status by Age for Women**. Average Income = mean monthly income per respondent. Low Income = 1 standard deviation below the mean or 0 if negative. High Income = 1 standard deviation above the mean. **b) Interaction of Partner Status by Age for Men**.

The interaction of partner status X age was significantly associated with depression severity for both women (B = 0.13, 95% CI: 0.02–0.25) and men (B = -0.08, 95% CI: -0.17 –-0.00), but the direction of the association was opposite for women versus men. As men age, having a partner is associated with lower depression severity, but as women age, having a partner is associated with high depression severity (Fig 3A and 3B).

## Discussion

This study demonstrates high rates of depression among a sample of 362 PLH at government sponsored ART clinics in Kolkata, India. Over 75% of the sample met the threshold for moderate or severe depression based on the HADS-D instrument. Previous studies found a depression prevalence ranging from 12% –40% among PLH in India [6, 43], and a 12% prevalence of major depression and 13% prevalence of other depression symptoms among PLH in the United States [44]. The depression rate in this study is likely higher than most previous India and United States samples because the sample in this study is very low income. As a result, the participants in this study may have many structural risk factors for depression beyond their HIV diagnosis and ART adherence challenges. This study is the first to our knowledge to investigate gender disparities in depression, internal HIV/AIDS stigma, social support, and illness coping strategies among ART patients in India. The findings also provide support for the gendered stress process model proposed.

Gender disparities in depression severity among PLH in India are created and maintained by systems of social stratification that disadvantages women compared to men. This study’s results suggest that women’s lower social status and limited access to resources contribute to higher severity of depression. These findings are consistent with a study conducted in Kolkata, India that found 38.3% of female sex workers suffered from depression, with 27.3% having moderate to severe depression [45]. Existing literature has shown that female sex workers’ depression may be associated with family- and partner-related burdens and lack of support from husbands [46, 47]. Expectations regarding caregiving roles based on gender likely exacerbate these disparities. For example, we would expect that marriage or cohabitation would provide protective effects that reduce the severity of depression for PLH as they age because partnered individuals have more ready access to social and emotional support within the household and potentially to additional income. It is documented in different contexts that the positive influence of adequate emotional support can reduce depression among women living with HIV [48, 49]. However, because women living with HIV in India are more likely to have an HIV-positive spouse than men living with HIV, women take on additional burdens and receive less support from partners.

Previous research has found the need for instrumental support is greater among men than women [50]. This relationship was substantiated by our finding that coping by drawing on instrumental support was protective for men at all income levels, but only protective for high-income women. For low-income women and women with no individual income, attempting to cope by frequently drawing on instrumental support increased the severity of depression. This effect is likely due to low-income women being embedded within resource-poor social networks that have very little support to readily provide. Frequently seeking instrumental support can deplete the few scarce resources available to low-income women for coping with all types of stressors, eroding both material and social support, as our proposed gendered stress process model suggests.

The results also suggest that as men age, having a partner reduces the severity of depression, but as women age, having a partner increases the severity of depression. The likely explanation is that women are providing care *to* their partners as they age but are not receiving sufficient care *from* their partners. One factor that may be contributing to this pattern is that the women in the sample are significantly more likely to have HIV-positive partners than the men in the sample. This is reflective of trends in HIV transmission in India, generally, because most women living with HIV who are not sex workers contract HIV from their husbands/intimate partners [51, 52], whereas men are more likely to contract HIV from someone other than their wives/intimate partners [53].

Internalized HIV/AIDS stigma was a strong predictor of depression severity in this sample. In line with our hypothesis, internalized HIV/AIDS was positively associated with depression level for both men and women. Although we hypothesized that exposure to stigma as a stressor would be higher among women and thus contributing to these disparities, we did not find support for this hypothesis. Our findings are consistent with the hypotheses that the conditional effects of lower social status and fewer access to resources for women leads to disparities in depression severity between women and men even though to the effect of stigma is similar for both women and men.

### Limitations

We acknowledge several limitations inherent to this study. First, the HADS-D is not a diagnostic measure of depression, but it is a reliable, brief assessment tool that is widely used in medical settings globally [54, 55], is highly correlated with diagnostic measures (i.e., has good concurrent validity), and has been translated in Bengali and validated for use in this context [35]. Second, depression was far higher than other samples. More than 90% of our sample reached the cutoff for at least mild depression based on the adapted HADS-D subscale. Two studies utilizing the HADS-D screening tool to measure depression among PLH found a depression prevalence ranging between 15%-30% [56, 57]. Several recent studies have found higher depression rates are associated with ART nonadherence compared to PLH who are adherent to their medication [58–60]. This may provide insight into the higher rates of depression among our sample of ART patients compared to other samples of PLH which may include people who are adherent to their ART regimens. We acknowledge this may not be a representative sample and results may not be generalizable to all PLH in India. Third, the cross-sectional nature of the analysis does not allow for causal inferences on temporality of variables examined. Therefore, it is important to be aware of the predictive limitations of the results. Since all variables are simultaneously assessed, it is difficult to infer temporal association between depression and psychosocial factors. In particular, the associations between behavioral disengagement and higher depression levels may be interpreted as the onset of depression preceded using behavioral disengagement to cope. Alternatively, PLH who report behavioral disengagement—or “giving up” attempts to cope with HIV and taking ART—may be at higher risk for developing depression over time [38]. Similarly, the findings surrounding self-distraction coping warrant further research. The observed association between self-distraction and lower depression severity may be interpreted that those with lower depression severity are able to take interest in things that distract from their HIV diagnosis, while those with more severe depression tend to lose interest in pleasurable activities. Previous literature has found self-distraction to be a maladaptive coping strategy resulting from depression [38, 61]. However, other studies found the use of self-distraction is associated with higher quality of life and lower levels of depression [62, 63]. The findings suggest the need for future longitudinal studies to better understand causal flow and/or bidirectional relationships of depression and psychosocial factors among PLH in India. The results of this cross sectional study may inform the hypotheses for a more complex, longitudinal cohort study.

## Conclusion

This study demonstrates high rates of depression among ART patients in Kolkata, India and identifies psychosocial factors associated with depression that may be targets of future interventions, including internalized HIV/AIDS stigma, social support, and important coping and self-management strategies, which vary by gender. The findings identify a significant need among PLH in India for programs or services targeting mental health, tailored by gender, age, and partner status. India’s National AIDS Control Organization (NACO) previously supported PLH through a comprehensive program that addressed positive living, psychosocial support, counseling, referrals, and linkage to needs-based services [64]. This effort was part of the national response to meet the needs of PLH, specifically those from high-risk groups, women, and children [64]. However, in a massive cleanup of corruption and inadequate staffing, NACO discontinued its support for these services in 2008 [65]. Social support programs should be reinstated as part of non-medical services for PLH in resource-poor settings, particularly to facilitate additional support systems for women infected with HIV [66]. There is a critical lack of mental health services globally, particularly in low and middle income countries [8]. Mental health services may not only improve quality of life and daily functioning, but also support ART adherence and retention. These findings can inform enhanced strategies in future interventions to address mental health, access to material and social resources, and ART adherence. Gender-specific tailoring of interventions will likely be needed to optimize intervention effects.
