# Correction: Gender disparities in depression severity and coping among people living with HIV/AIDS in Kolkata, India

**DOI:** 10.1371/journal.pone.0213093

**Published:** 2019-02-22

**Authors:** Dallas Swendeman, Anne E. Fehrenbacher, Soma Roy, Rishi Das, Protim Ray, Stephanie Sumstine, Toorjo Ghose, Smarajit Jana

There are errors in the caption for Fig 2. Please see the complete, correct Fig 2 caption here.

There are errors in the caption for Fig 3. Please see the complete, correct Fig 3 caption here.

**Fig 2:**
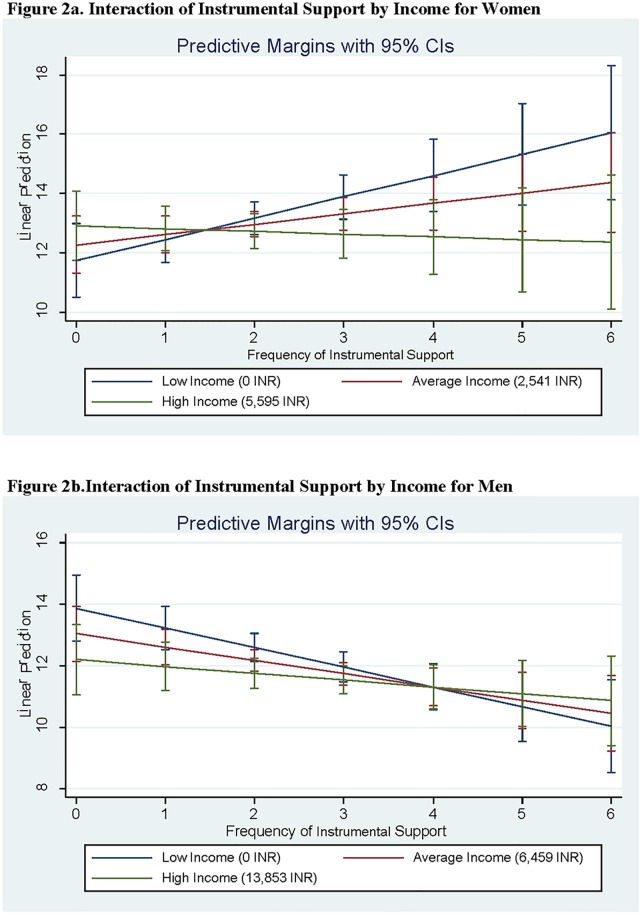
**a) Interaction of Instrumental Support by Income for Women**. **b) Interaction of Instrumental Support by Income for Men**. Average Income = mean monthly income per respondent. Low Income = 1 standard deviation below the mean or 0 if negative. High Income = 1 standard deviation above the mean.

**Fig 3:**
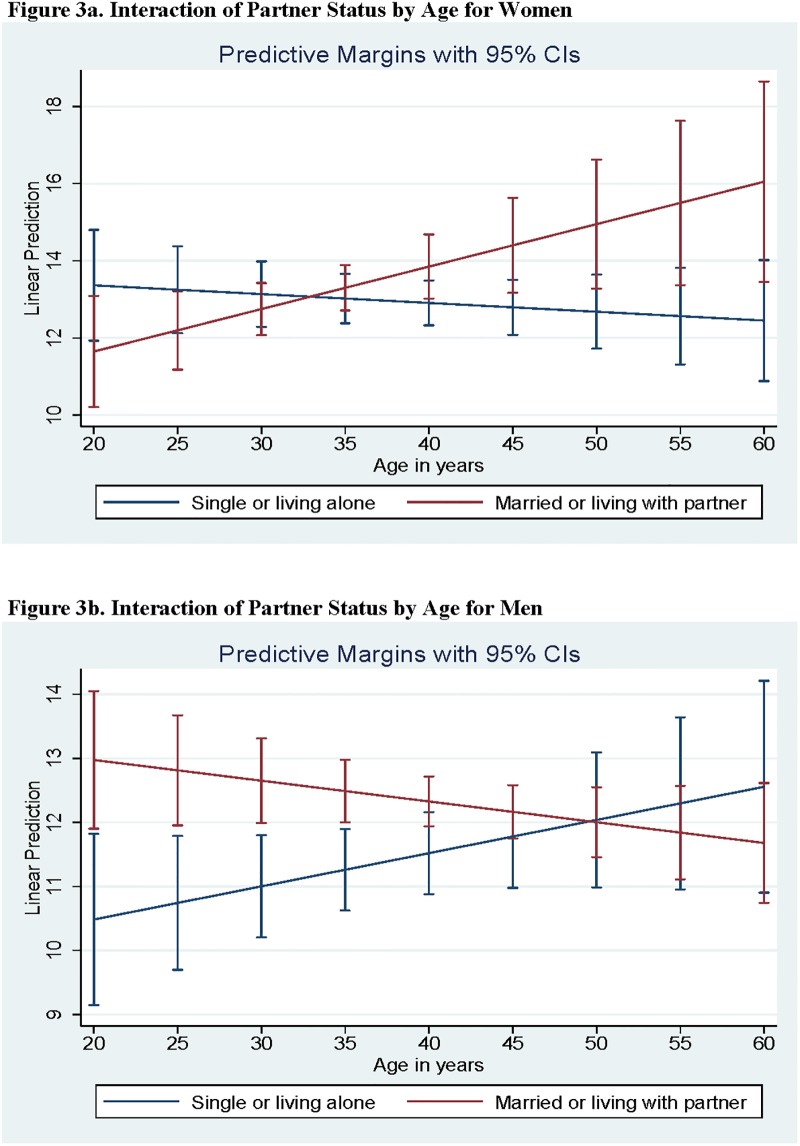
a) Interaction of Partner Status by Age for Women. b) Interaction of Partner Status by Age for Men.
